# Hyperphosphorylated Tau and Cognition in Epilepsy

**DOI:** 10.3390/jcm14020514

**Published:** 2025-01-15

**Authors:** Juri-Alexander Witt, Johanna Andernach, Albert Becker, Christoph Helmstaedter

**Affiliations:** 1 Department of Epileptology, University Hospital Bonn (UKB), 53127 Bonn, Germany; s45jande@uni-bonn.de (J.A.); christoph.helmstaedter@ukbonn.de (C.H.); 2 Section for Translational Epilepsy Research, Institute of Neuropathology, Medical Faculty, University of Bonn, 53127 Bonn, Germany; umt913@uni-bonn.de

**Keywords:** epilepsy, seizures, tau, hyperphosphorylation, neurodegeneration, hippocampus, temporal cortex, neuropsychology, cognition, dementia

## Abstract

In light of the growing interest in the bidirectional relationship between epilepsy and dementia, this review aims to provide an overview of the role of hyperphosphorylated tau (pTau) in cognition in human epilepsy. A literature search identified five relevant studies. All of them examined pTau burden in surgical biopsy specimens from patients with temporal lobe epilepsy. The prevalence of pTau reported across the five studies, encompassing a total of 142 patients, ranged from 3.5% to 95%. Findings also varied regarding the location of pTau in the hippocampus and/or temporal cortex. Two of five studies (40%) demonstrated an inverse relationship between pTau burden and cognitive performance, one study with regard to executive functions and the other with regard to naming and verbal short-term memory. The only longitudinal study found a significant link between pTau and cognitive decline in verbal learning and memory, and in part also in naming, from the pre- to the postoperative assessment and from three to 12 months postoperatively. Given the heterogeneity of the study cohorts and the neuropsychological and neuropathological methodologies and findings, no clear picture emerges regarding the association between pTau and cognition in temporal lobe epilepsy. Added to this is the multifactorial etiology of cognitive impairment in epilepsy, including the active epilepsy, the underlying and sometimes dynamic pathology, and anti-seizure medication. Some of these factors may affect pTau expression. Further research should aim to investigate pTau longitudinally and noninvasively on a whole-brain level, using targeted neuropsychological outcome measures and controlling for age and other factors potentially influencing cognitive trajectories in epilepsy.

## 1. Introduction

A prominent topic of current interest in the field of epilepsy is the role of hyperphosphorylated tau (pTau) for neuronal damage, the disruption of neuronal network function, and the clinical manifestation of cognitive impairment. PTau is discussed as a potential connecting link between epilepsy, dementia, and neurodegenerative diseases [1,2,3].

Tau is a protein essential for stabilizing microtubules. The hyperphosphorylation of tau (in the form of neuropil threads, pre-tangles, or neurofibrillary tangles) leads to a loss of this function, resulting in the collapse of the axonal microtubules. Consequently, pTau may disrupt axonal transport and ultimately cause neuronal dysfunction. The accumulation of pTau in the brain is also linked to epilepsy and network hyperexcitability observed across various neurological disorders [4]. In this focused review, we summarize studies investigating the effects of pTau on cognition in individuals with epilepsy.

## 2. Materials and Methods

We conducted a literature search in September 2024 using PubMed with the following search term: tau[title] AND epilepsy[title] AND (cogn* OR memory). The inclusion criteria required original studies published in peer-reviewed journals that assessed pTau and objective cognitive performance in individuals with epilepsy. The search was limited to English articles. The literature search was performed by J.A. and validated by J.-A.W.

## 3. Research Status on Hyperphosphorylated Tau and Cognition

The literature search yielded 13 hits. Six of these papers were reviews, one was an original study on animal research, and one was not on cognition, so the search identified a total of five relevant studies on pTau and cognition in individuals with epilepsy. The major study characteristics are summarized in Table 1.

The first relevant study by Tai et al. [5] from the UK, published in 2016, was based on neuropathological examinations of specimens from 33 patients who had undergone epilepsy surgery for mesial temporal lobe epilepsy. The authors hypothesized that pTau burden would be correlated with cognitive decline in temporal lobe epilepsy. The age at surgery ranged from 50 to 65 years with a mean of 53.6 years. The average age at epilepsy onset was 14.7 years (no range reported). All patients had hippocampal sclerosis according to the neuropathological examination. PTau was identified via immunohistochemistry (AT8) and quantified applying a modified tau score devised specifically for the analysis of specimens from anterior temporal lobe resections. The prevalence of pTau (including neuropil threads, pre-tangles, and neurofibrillary tangles) was very high, i.e., 31/33 cases (94%). However, there was a relative sparing of the sclerotic hippocampus. Cognitive data (verbal and visual learning and memory, naming, and phonemic and semantic fluency; the employed tests are listed in Table 2) gathered before as well as 3 and 12 months after surgery were available in a subgroup of 21 patients (64%). In addition, intelligence was measured before surgery. The analysis focused on cognitive changes from pre- to 12 months postoperative and from 3 to 12 months postoperative. First of all, there was no significant association between pTau burden and presurgical neuropsychological performance. At the 12-month follow-up, a significant deterioration in verbal memory and a discrete improvement in visual memory were observed compared to the presurgical status. There was a significant association between this cognitive change and the pTau burden. The higher the latter was, the greater the cognitive decline was. This was especially valid for verbal learning (r = −0.63), followed by naming performance (r = −0.50) and verbal memory (r = −0.44). When analyzing the cognitive change from the early to the later postoperative follow-up, the same association was solely found for verbal learning (r = −0.54). The authors concluded that pTau contributes to an accelerated decline of cognitive functions in epilepsy.

Gourmaud and colleagues [6] from the US also investigated tissue gathered from anterior temporal lobe resections from a much younger cohort of 19 patients with phamacoresistant epilepsy. The age at surgery ranged from 10 to 56 years with an average of 29 years. The mean age at epilepsy onset was 14.8 years (calculated based on Supplementary Table S1 of [6]), ranging from 1 to 36 years. The most frequent primary pathology was hippocampal sclerosis (63.2%), with few patients exhibiting dual pathology (including FCD type IIa, dysembryoplastic neuroepithelial tumor, periventricular heterotopia) and one patient with leptomeningeal vascular malformation. Besides the presence of Aβ in hippocampal tissue, immunohistochemical analysis revealed an increased expression of pTau in both the hippocampus and temporal neocortex. The cognitive analysis focused on 14 adult patients (74%) and the presurgical assessment of intelligence (Wechsler Adult Intelligence Scale IV, WAIS IV), executive functions (digit span backward test; Trail Making Test B, TMT-B), verbal (California Verbal Learning Test II, CVLT-II) and visuospatial long-term memory (BVMT-R), visuospatial functions (Brief Visuospatial Memory Test Revised, BFRT; Rey–Osterrieth Complex Figure Test, ROCF), language (naming subtest of Neuropsychological Assessment Battery, NAB), and dexterity (Grooved Pegboard Test, GPT). Both total and pTau (as well as elements of the amyloid signaling pathway) correlated with deficits in executive functions (processing speed efficiency and verbal working memory). The authors concluded that amyloid- and tau-associated neurodegeneration similar to Alzheimer’s disease may contribute to cognitive impairment in patients with drug-refractory temporal lobe epilepsy.

Silva et al. [7] from Australia analyzed resective specimens from 56 patients who had undergone epilepsy surgery for drug-resistant temporal lobe epilepsy. The age at anterior temporal lobectomy ranged from 20 to 68 years with a median of 34 years. The age at epilepsy onset varied from 0 to 56 years (median: 15). Histology revealed hippocampal sclerosis in the majority of patients (51.9%). The prevalence of pTau (3.5%) and Aβ plaques (7%) was low. PTau in form of neurofibrillary tangles was only seen in the temporal cortex and not in the hippocampus. The presurgical neuropsychological assessment included measures of verbal (Rey Auditory Verbal Learning Test (RAVLT), Verbal Pair Associates (VPA) subtest from Wechsler Memory Scale Revised (WMS-R)) and visual memory (Rey–Osterrieth Complex Figure Test (ROCF)). Depending on the test, cognitive data were available from 31 (55%) to 52 patients (93%). Patients with Aβ plaques scored worse in the more demanding part of the VPA subtest. There were no other significant associations between presurgical memory performance and Aβ plaques or pTau. Given the low prevalence of pTau and Aβ plaques, the authors stated that “it is unlikely that cognitive impairment in TLE is driven by the same mechanisms as in Alzheimer disease” (p. 3058).

The study by Toscano and colleagues [8] from the US and Brazil in 2023 determined pTau in the resected hippocampal formation of 22 patients who had undergone epilepsy surgery for mesial temporal lobe epilepsy. The age ranged from 30 to 58 years with a mean of 41.8 years. The age at epilepsy onset was rather early with an average of 4.3 years (range: 0–13 years). In all patients, hippocampal sclerosis was diagnosed via histology. Compared to the non-sclerotic hippocampi of 20 autopsy controls of comparable age, the hippocampal pTau burden was significantly higher in patients with epilepsy. PTau was detected in 95% of the hippocampal specimens. Among the patients, those with hippocampal sclerosis type 2 had a higher pTau burden than those with type 1. Aβ deposits were not observed. The presurgical neuropsychological examination which was performed in all included 22 patients focused on verbal short-term memory (digit span of the Wechsler Adult Intelligence Scale (WAIS)), verbal and visual long-term memory (Logical Memory I and II, Visual Reproduction I and II of the Wechsler Memory Scale (WMS)), and confrontative naming (Boston Naming Test (BNT)). A higher pTau burden in the hippocampus was associated with deficits in confrontative naming and verbal memory span. In a multivariate model adjusted for age, sex, education, seizure frequency, and the type of hippocampal sclerosis, only the verbal memory span showed still an association with the pTau burden. The authors concluded that tau pathology may be a potential contributor to cognitive impairment in mesial temporal lobe epilepsy.

The most recent study that analyzed surgical biopsy specimens was published in 2023 by Aroor et al. [9] from the US. The authors determined pTau levels (along with Aβ) in the resective specimens from temporal lobe resections performed in 12 patients with drug-resistant epilepsy. The age ranged from 24 to 67 years with an average of 42.5 years. The mean age at epilepsy onset (calculated based on Table 1 of [9]) was 18.9 years (range: 1–66). The actual primary pathologies were unfortunately only reported for two patients (FCD type Ib and FCD type IIIa); for the others, only presumptive MRI findings were listed including mesial temporal sclerosis, cortical dysplasia, heterotopia, possible neoplasm, and three non-specific cases. Immunohistology and enzyme-linked immunoassays were limited to biopsies from the temporal cortex (hippocampi were not analyzed) and indicated a robust presence of pTau in the form of neuropil threads and neurofibrillary tangles in 50% of the patients. Aβ deposits were present in 67% of the cases. Cognitive data were limited to the presurgical results of an abbreviated Wechsler intelligence test which were only available for eight of the twelve patients (67%). Correlation analyses revealed no significant associations between pTau levels at two different sites (Thr181 and Thr205) and intelligence. However, the non-significant positive (!) correlation coefficient was large at site Thr181 (r = 0.54; *p* = 0.16) which would indicate a higher intelligence with a higher pTau burden. The non-significance rather points to a low statistical power due to the low sample size. Thus, the study confirms that pTau (as well as Aβ deposits) can be found in patients with drug-refractory temporal lobe epilepsy, but the association with global cognitive performance remains somewhat unclear.

## 4. Synthesis and Discussion

All the five presented studies analyzed pTau in the surgical biopsy specimens of patients who had undergone epilepsy surgery for drug-refractory temporal lobe epilepsy. The prevalence of pTau ranged from 3.5 to 95%. The lowest prevalence was found in the largest study [7]. The findings on the relationship between pTau and neuropsychological performance were heterogeneous. While all studies analyzed presurgical cognitive performance, only two out of five (40%) found an inverse association with pTau burden, with one study regarding executive function [6] and the other concerning language function (i.e., naming) and verbal short-term memory [8]. Naming is associated with the temporal lobe (via the ventral stream also known as the “what pathway”) [10], whereas executive function and verbal short-term memory are rather dependent on an extratemporal fronto-parietal network [11,12]. Consequently, a relationship of the latter functions with pTau in temporal and/or hippocampal tissue is unexpected. However, one potential explanation is that a more widespread brain involvement exists, but the chosen methodology is limited to demonstrating pTau only in the resected tissue of the temporal lobe (i.e., a sampling bias). Post-mortem studies as published by Thom and colleagues [13] may clarify pTau distribution in the brain. Furthermore, in vivo approaches, such as positron emission tomography (PET) [14], are needed to assess pTau comprehensively.

Solely one study [5] also analyzed longitudinal cognitive data, finding inverse relationships between pTau and a decline in verbal learning and long-term memory as well as in naming. However, this study found no association with presurgical performance. The largest study by Silva et al. [7] reported no significant correlation between pTau and presurgical memory performance, but it must be considered that this study also found the lowest prevalence of pTau.

Overall, no consistent relationship between pTau and cognition in temporal lobe epilepsy emerges. In this respect, the apparent heterogeneity across the studies regarding the demographic characteristics of the study cohorts (Table 1) as well as the neuropathological (Table 3) and the neuropsychological methodology (Table 2) needs to be discussed. The sample sizes ranged from 12 to 56 patients with a total number of 142 patients. Three of the studies included fewer than 30 patients. When analyzing the relationship between pTau and neuropsychological performance (Table 2), sample sizes dropped by up to 45% because of missing data. So, the main analyses are based on sample sizes ranging from 8 to 52 patients (total: 96–117 patients). The average patient age varied from 34 to 54 years, with a total range of 10–68 years. The age at epilepsy onset ranged from 0 to 66. Therefore, elderly patients with late new-onset epilepsy (≥50 years) appear to be very rare. Regarding neuropathological approaches, one study exclusively analyzed the hippocampal formation [8], whereas one study only focused on the temporal cortex [9]. Three studies examined both locations: one found pTau only in the temporal cortex [7], another mostly in the temporal cortex [5], and one in both the hippocampus and temporal cortex [6]. Mixed pathologies complicate findings, with tauopathy potentially linked to primary lesions [15]. Neuropsychological methods showed substantial heterogeneity in cognitive domains and test instruments. The studies addressed one to six cognitive domains which were assessed via a total of one to nine tests. In the light of temporal lobe surgery, the most frequently addressed cognitive domain was (material-specific) episodic long-term memory (four out of five studies) followed by language (three out of five studies) and intelligence (three out of five studies). Each of the studies assessing material-specific long-term memory employed different tests, while intelligence tests were all derivatives of the WAIS. The heterogeneity of neuropsychological tests aligns with national and international surveys on neuropsychological practice in epilepsy centers [16,17,18]. However, tests can differ in their validity to assess temporal and hippocampal dysfunction [19,20,21] and thus be more or less suited to demonstrate a potential relationship between pTau and memory performance.

Finally, it is important to underscore that the etiology of cognitive deficits in epilepsy is multifactorial [22,23]. Potential effects of the active epilepsy (epileptic seizures and interictal epileptic discharges), the underlying pathology, antiseizure medication, and psychiatric comorbidities need to be taken into consideration along with compensatory mechanisms (reserve capacities, plasticity) and neurodevelopmental aspects (developmental hindrance, senescence).

In a recent study [24], an in-depth reanalysis of resective specimens from patients who had undergone epilepsy surgery and showed an unexpected cognitive decline in the time thereafter in addition to any direct surgical effects was performed. First of all, with 30/355 patients (8%), unexpected cognitive deterioration was rare. Among the 24 patients with available specimens, 71% had further neuropathological changes in addition to the typical spectrum (such as hippocampal sclerosis, focal cortical dysplasias, etc.), including a secondary, putatively epilepsy-independent neurodegenerative disease process, limbic inflammation or “hippocampal gliosis” without segmental neurodegeneration. PTau was found in only three of the patients (12.5%) and solely in temporal specimens. It is important to note that cognitive decline is not typical in chronic epilepsy [25,26] and should always prompt a thorough investigation of the underlying cause [24,27].

## 5. Conclusions

The role of pTau in cognitive dysfunction and decline has emerged as a topic of significant interest in the field of epilepsy, since pTau may provide a link between epilepsy and dementia. However, the available studies which evaluated pTau burden in resected specimens from patients with drug-refractory temporal lobe epilepsy report inconsistent findings. This variability may stem from heterogeneity in the demographic characteristics of the patient cohorts, as well as differences in the neuropathological and neuropsychological methodologies. Another challenge lies in the multifactorial etiology of cognitive impairments in people with epilepsy. Future studies should focus on quantifying pTau burden in vivo at the whole-brain level. Additionally, employing evidence-based cognitive measures to examine the potential association with pTau, while accounting for critical cognition-related factors, would enhance the robustness of studies.

## Figures and Tables

**Table 1:** Major study characteristics including year of publication, origin, total sample size, chronological age, age at epilepsy onset, and, if applicable, information on controls.

	Year of Publication	Country	N	Age, Years (Range)	Age At Epilepsy Onset, Years (Range)	Controls	References
1	2016	UK	33	54 (50–65)	15 (n.a.)	Age-matched population controls from a post-mortem series	[5]
2	2020	US	19	29 (10–56)	15 (1–36)	22 neurologically normal and 9 Alzheimer’s disease autopsy cases	[6]
3	2021	AUS	56	34 (20–68)	15 (0–56)	-	[7]
4	2023	US/BR	22	42 (30–58)	4 (0–13)	20 hippocampi of neurologically normal autopsy cases	[8]
5	2023	US	12	43 (24–67)	19 (1–66)	-	[9]

n.a., not available; UK, United Kingdom; US, United States of America; AUS, Australia; BR, Brazil.

**Table 2:** The neuropsychological methods and findings of the reviewed studies. The table summarizes the assessed cognitive functions and tests, the number of the assessed patients in relation to the total sample, and major findings regarding the relationship between pTau burden and neuropsychological performance.

	Assessed Cognitive Functions and Tests	N	Relationships Between pTau and Objective Neuropsychological Performance	References
1	Intelligence (WAIS), verbal and visual learning and long-term memory (AMIPB or BIMPB), naming (GNT), and phonemic and semantic fluency	21/33 (64%)	There was no significant association between pTau and presurgical neuropsychological performance. A higher pTau burden was inversely correlated with cognitive decline from pre- to 12 months postoperative (verbal learning (r = −0.63) and memory (r = −0.44) and naming (r = −0.50)) as well as from 3 months to 12 months postoperative (verbal learning (r = −0.54)).	[5]
2	Intelligence (WAIS IV), executive function (digit span backward test, TMT-B), verbal (CVLT-II) and visuospatial long-term memory (BVMT-R), visuospatial functions (BFRT, ROCF), language (naming subtest of NAB), and dexterity (GPT)	14/19 (74%)	Both the total and pTau burden inversely correlated with presurgical deficits in executive functions (processing speed efficiency (r = −0.78) and verbal working memory (r = −0.89)).	[6]
3	Verbal learning and long-term memory (RAVLT, VPA from WMS-R) and visual long-term memory (ROCF)	31–52/56 (55–93%)	No significant correlation between pTau and presurgical memory performance was found.	[7]
4	Verbal short-term memory (subtest of WAIS), verbal (LM I and II of WMS) and visual long-term memory (VR I and II of WMS), and confrontative naming (BNT)	22/22 (100%)	A higher pTau burden was associated with deficits in confrontative naming and verbal memory span. The latter relationship was confirmed by a multivariate model adjusted for age, sex, education, seizure frequency, and the type of hippocampal sclerosis.	[8]
5	Intelligence (abbreviated WAIS)	8/12 (67%)	No significant correlation between pTau levels at two different sites (Thr181 and Thr205) and intelligence was found. However, the non-significant positive (!) correlation coefficient was large at site Thr181 (r = 0.54; *p* = 0.16).	[9]

AMIPB, Adult Memory and Information Processing Battery; BFRT, Benton Facial Recognition Test; BIMPB, BIRT Memory and Information Processing Battery; BNT, Boston Naming Test; BVMT-R, Brief Visuospatial Memory Test Revised; CVLT-II, California Verbal Learning Test II; GNT, Graded Naming Test; GPT, Grooved Pegboard Test; LM, Logical Memory; pTau, hyperphosphorylated tau; NAB, Neuropsychological Assessment Battery; RAVLT, Rey Auditory Verbal Learning Test; ROCF, Rey–Osterrieth Complex Figure Test; TMT-B, Trail Making Test B, VPA, Verbal Paired Associates; WAIS, Wechsler Adult Intelligence Scale; WMS(-R), Wechsler Memory Scale (Revised).

**Table 3:** Neuropathological methods and findings including prevalence and form of pTau.

	N	Hippocampal Sclerosis	Method (pTau)	Prevalence of pTau	Form of pTau	Location	Other Findings	References
1	33	100%	IHC (AT8)	94%	NT + PT + NFT	Mostly TC; relative sparing of HC	Aβ plaques in 15%	[5]
2	19	63%	IHC (AT8, clone Tau5)	n.a.	n.a.	TC + HC	APP deposits in 3/11 (27%)	[6]
3	56	52%	IHC (AT8)	3.5%	NFT	TC, not HC	Aβ plaques in 7%	[7]
4	22	100%	IHC + MA	95%	NT + PT + NFT	HC (TC n.a.)	Aβ deposits absent	[8]
5	12	n.a.	IHC + IA	50%	NT + NFT	TC (HC not analyzed)	Aβ deposits in 67%	[9]

n.a., not available; Aβ, amyloid beta; APP, amyloid precursor protein; HC, hippocampus; HS, hippocampal sclerosis; IHC, immunohistochemistry; IA, immunoassays; MA, morphometric analyses; NFT, neurofibrillary tangles; NT, neuropil threads; PT, pre-tangles; pTau, hyperphosphorylated tau; TC, temporal cortex.

## Data Availability

All data are presented in the article and tables.

## References

[1] Sen A., Capelli V., Husain M. (2018). Cognition and Dementia in Older Patients with Epilepsy. Brain.

[2] Sánchez M., García-Cabrero A., Sánchez-Elexpuru G., Burgos D., Serratosa J. (2018). Tau-Induced Pathology in Epilepsy and Dementia: Notions from Patients and Animal Models. Int. J. Mol. Sci..

[3] Hickman L.B., Stern J.M., Silverman D.H.S., Salamon N., Vossel K. (2023). Clinical, Imaging, and Biomarker Evidence of Amyloid- and Tau-Related Neurodegeneration in Late-Onset Epilepsy of Unknown Etiology. Front. Neurol..

[4] Hwang K., Vaknalli R.N., Addo-Osafo K., Vicente M., Vossel K. (2022). Tauopathy and Epilepsy Comorbidities and Underlying Mechanisms. Front. Aging Neurosci..

[5] Tai X.Y., Koepp M., Duncan J.S., Fox N., Thompson P., Baxendale S., Liu J.Y.W., Reeves C., Michalak Z., Thom M. (2016). Hyperphosphorylated Tau in Patients with Refractory Epilepsy Correlates with Cognitive Decline: A Study of Temporal Lobe Resections. Brain.

[6] Gourmaud S., Shou H., Irwin D.J., Sansalone K., Jacobs L.M., Lucas T.H., Marsh E.D., Davis K.A., Jensen F.E., Talos D.M. (2020). Alzheimer-like Amyloid and Tau Alterations Associated with Cognitive Deficit in Temporal Lobe Epilepsy. Brain.

[7] Silva J.C., Vivash L., Malpas C.B., Hao Y., McLean C., Chen Z., O’Brien T.J., Jones N.C., Kwan P. (2021). Low Prevalence of Amyloid and Tau Pathology in Drug-Resistant Temporal Lobe Epilepsy. Epilepsia.

[8] Toscano E.C.B., Vieira É.L.M., Grinberg L.T., Rocha N.P., Brant J.A.S., Paradela R.S., Giannetti A.V., Suemoto C.K., Leite R.E.P., Nitrini R. (2023). Hyperphosphorylated Tau in Mesial Temporal Lobe Epilepsy: A Neuropathological and Cognitive Study. Mol. Neurobiol..

[9] Aroor A., Nguyen P., Li Y., Das R., Lugo J.N., Brewster A.L. (2023). Assessment of Tau Phosphorylation and β-Amyloid Pathology in Human Drug-Resistant Epilepsy. Epilepsia Open.

[10] Drane D.L., Pedersen N.P. (2019). Knowledge of Language Function and Underlying Neural Networks Gained from Focal Seizures and Epilepsy Surgery. Brain Lang..

[11] Markowitsch H.J., Kalbe E., Kessler J., Von Stockhausen H.M., Ghaemi M., Heiss W.D. (1999). Short-Term Memory Deficit after Focal Parietal Damage. J. Clin. Exp. Neuropsychol..

[12] Müller N.G., Knight R.T. (2006). The Functional Neuroanatomy of Working Memory: Contributions of Human Brain Lesion Studies. Neuroscience.

[13] Thom M., Liu J.Y.W., Thompson P., Phadke R., Narkiewicz M., Martinian L., Marsdon D., Koepp M., Caboclo L., Catarino C.B. (2011). Neurofibrillary Tangle Pathology and Braak Staging in Chronic Epilepsy in Relation to Traumatic Brain Injury and Hippocampal Sclerosis: A Post-Mortem Study. Brain A J. Neurol..

[14] Bollack A., Pemberton H.G., Collij L.E., Markiewicz P., Cash D.M., Farrar G., Barkhof F. (2023). On behalf on the AMYPAD consortium Longitudinal Amyloid and Tau PET Imaging in Alzheimer’s Disease: A Systematic Review of Methodologies and Factors Affecting Quantification. Alzheimer’s Dement..

[15] Sarnat H.B., Flores-Sarnat L. (2015). Infantile Tauopathies: Hemimegalencephaly; Tuberous Sclerosis Complex; Focal Cortical Dysplasia 2; Ganglioglioma. Brain Dev..

[16] Witt J.-A., Helmstaedter C. (2009). Neuropsychologie Bei Epilepsie Teil II: Gibt Es Eine Gemeinsame Basis Zur Etablierung Diagnostischer Leitlinien?. Fortschritte Neurol. Psychiatr..

[17] Vogt V.L., Äikiä M., del Barrio A., Boon P., Borbély C., Bran E., Braun K., Carette E., Clark M., Cross J.H. (2017). Current Standards of Neuropsychological Assessment in Epilepsy Surgery Centers across Europe. Epilepsia.

[18] Djordjevic J., Jones-Gotman M., Helmstaedter C., Hermann B., Lassonde M., Kahane P., Arzimanoglou A. (2011). Inquiry on Assessments across Epilepsy Centers in Different Countries. Neuropsychology in the Care of People with Epilepsy.

[19] Witt J.-A., Meschede C., Helmstaedter C. (2021). Hazardous Employment of Invalid Measures for Cognitive Outcome Assessment: You Only See What Your Test Can Show You. Epilepsy Behav..

[20] Helmstaedter C., Wietzke J., Lutz M.T. (2009). Unique and Shared Validity of the “Wechsler Logical Memory Test”, the “California Verbal Learning Test”, and the “Verbal Learning and Memory Test” in Patients with Epilepsy. Epilepsy Res..

[21] Loring D.W., Strauss E., Hermann B.P., Barr W.B., Perrine K., Trenerry M.R., Chelune G., Westerveld M., Lee G.P., Meador K.J. (2008). Differential Neuropsychological Test Sensitivity to Left Temporal Lobe Epilepsy. J. Int. Neuropsychol. Soc..

[22] Helmstaedter C., Witt J.-A. (2012). Clinical Neuropsychology in Epilepsy: Theoretical and Practical Issues..

[23] Witt J.A., Becker A.J., Helmstaedter C. (2023). The Multifactorial Etiology of Cognitive Deficits in Epilepsy and the Neuropathology of Mesial Temporal Lobe Epilepsy beyond Hyperphosphorylated Tau. Alzheimer’s Dement..

[24] Reimers A., Helmstaedter C., Elger C.E., Pitsch J., Hamed M., Becker A.J., Witt J.A. (2022). Neuropathological Insights into Unexpected Cognitive Decline in Epilepsy. Ann. Neurol..

[25] Helmstaedter C., Elger C.E. (2009). Chronic Temporal Lobe Epilepsy: A Neurodevelopmental or Progressively Dementing Disease?. Brain A J. Neurol..

[26] Helmstaedter C., Elger C.E. (1999). The Phantom of Progressive Dementia in Epilepsy. Lancet.

[27] Helmstaedter C., Lutz T., Wolf V., Witt J.A. (2023). Prevalence of Dementia in a Level 4 University Epilepsy Center: How Big Is the Problem?. Front. Neurol..

